# Does the National Level Development Zone foster cities’ innovation capacity? A study from China

**DOI:** 10.1371/journal.pone.0341573

**Published:** 2026-02-06

**Authors:** Luping Bao

**Affiliations:** Business School, China University of Political Science and Law, Beijing, People’s Republic of China; USTC: University of Science and Technology of China, CHINA

## Abstract

The article finds that the National Level Development Zone (NLDZ) development has affected urban innovation capacity using panel data from 2014 to 2022 in China. Compared to the West, the East NLDZ of China has a greater effect, and the NLDZ in the Northeast and the Middle Part have the least influence on urban innovation. However, the NLDZ influence of a city at the province level is greater than that at the prefecture level, and the effect of NLDZ on the Bond Area is the greatest, followed by that of the Economic and Technological Development Zone, while the impact of the High-Tech Industrial Development Zone is the least. Finally, we advise preserving a specific quantity of NLDZ to prevent the effect of declining marginal utility.

## Introduction

### Background

In China, the term “National Level Development Zone" (NLDZ) refers to the various categories of special economic zones, such as National Level Bond Area (BA), National Level Tourism Resort (TR), National Level Economic and Technological Development Zone (ETDZ), and National Level High-Tech Industrial Development Zone (HTIDZ), all which the State Council has approved to implement special preferential policies of the state [1,2]. NLDZ seeks to advance global commerce, high-tech industry development, and economic progress [1,3]. According to the China Development Zone Review and Announcement Catalogue (2018), there are a total of 552 in China. More specifically, ETDZ attracts foreign investment to support local economic development and is home to numerous foreign businesses [4]. But presently, ETDZ comprises many high-tech sectors, and its economic conditions are changing; its purpose also involves supporting the innovation of relevant industrial chains. HTIDZ is focused on industrial development [5]. Early industrial development needs protection and encouragement before it becomes stronger, and then seizes the market, or defeats foreign businesses. The Chinese government attaches significant priority to HTIDZ and formulates its scope and strategy to guide its diverse development [6]. For instance, government guidelines specify how much tax revenue should be allocated to foster high-tech industries in various cities, aiming to support them [7,8]. Like a free trade zone for other nations, the BA is the area authorized by the State Council to conduct bonded business and international trade [9]. These years, BA permits foreign money to own or operate export, bonded warehousing, and global trade companies [10]. To foster collaboration between domestic and overseas organizations, it also aims to spearhead innovative business models and leverage foreign innovation laboratories [11,12]. BA is becoming increasingly important to a city’s creative capacity. In addition to these special economic zones, the TR and the National Border Economic Cooperation Zone (BECZ) also promote economic growth and local innovation in distinct ways [13]. The former attracts tourists from other locations and advances tourism technology. In contrast, the latter attracts industries from bordering countries and national export businesses to boost tax income and create connections between local and foreign technology to enhance innovation capacity [14].

### The inquiry and approach

In China’s current economic conditions, we know that a country’s innovation capacity determines whether it can win in increasingly fierce international competition [15]. As an important economic booster, NLDZ’s GDP is up to 1/4 of China’s (Data from the official websites of the Ministry of Commerce and the Ministry of Science and Technology). So, it’s reasonable to ask whether NLDZ is helping foster cities’ innovation capabilities. After all, the unique innovations made by each city make up the nation’s innovation capacity.

We did not find relevant research on this topic in the Web of Science. It’s the marginal contribution of this literature. Then we explore the relationship between the development of NLDZ and cities’ innovation ability through a panel data set from 2014 to 2022. The results show that the development of NLDZ significantly enhances cities’ innovation capacity, and its impact in the East is greater than in the West, with the Middle Part and Northeast affected least.

### Following section

The following section is structured as follows: The next section provides a literature review; the Methods section describes the research methodology; the Results section presents the findings of the empirical study; the Discussion section analyzes the results; and finally, the Conclusion summarizes the article.

## Literature review

### The contribution of the National Level Development Zone

The articles on the effects of NLDZ discuss several aspects, including human capital development, cities’ green innovation capacity, economic growth, and production efficiency.

Hu, Huang [16] use differences-in-difference (DID) analysis to investigate the impact of China’s special economic zones on the accumulation of human capital. Their findings indicate that the programs implemented by special economic zones greatly increase the number of years people spend in school, with a focus on females and senior education levels. This article extends the research scope from the national to the provincial level and employs econometric methods in a relatively comprehensive manner. It did not, however, specify which school is located in a special economic zone or take the institution’s founding date into account. Specifically, the establishment date of each special economic zone ought to be taken into account, which is why the differences-in-differences approach is insufficient to address this issue [17]. Drawing on the analysis above, our study uses the economic scale of NLDZ as an explanatory variable rather than the policy implementation year.

Liu, Zhang [18] also examine whether the special economic zones promote green technology innovation in China using the differences-in-differences method. The results show that the special economic zone policy significantly improves enterprises’ green technology innovation. In the meantime, it demonstrates, through mechanism testing, that the enhancement of green technology innovation in enterprises results from both short-term preferential policy subsidies and long-term agglomeration effects. The paper uses a variety of research methods, appropriately defines firm coordination, and offers vital data on concerns about the nation. The difference from our work is that we research the whole innovation dimension of cities using patent counts. In contrast, Liu’s article does not examine the timing of special economic zone formation, which may lead to misleading results.

According to Karlina, Kovalenkova [19], living in a special economic zone can drastically lower a resident’s production costs, increasing the competitiveness of ship equipment and component manufacturing. This paper provides a thorough examination of how the Special Economic Zone ’Lotus’ stimulates innovation and economic growth in the Astrakhan region, with particular attention to the shipbuilding sector. However, the summary doesn’t make clear how the data were gathered and processed, which is crucial for assessing the reliability and validity of the results.

Above all, our work emphasizes the cities’ ability to innovate. As carriers of economic development, cities’ innovation capacity also directly affects the country’s innovation strength. This paper examines the effect of NLDZ development on urban innovation capacity, a topic rarely discussed in the existing literature.

### The characteristics and influence of NLDZ

Panfilova, Tikhonov [20] use analytical materials, statistical techniques, and comparative and system research approaches to investigate the competitive advantages of high-tech manufacturers in special economic zones. The findings demonstrate that the competitive advantages of high-tech businesses in special economic zones result from agglomeration effects, enterprise management expertise, and advantageous tax policies. The paper rates the attractiveness of special economic zones using a thorough index; however, it lacks empirical support, which could make it less credible. In Wang, Lin [21], the relationship between industrial clustering and technological innovation is primarily discussed, with a focus on the Information and Communication Technology industry in Shenzhen, China. The study addresses the intense inquiry and debate over this relationship. The analysis found no connection between spatial agglomeration and national economic performance. Although the research is based on complete data from a national economic census, which provides a rigorous empirical basis for the analysis, it may not be broadly applicable to various types of industrial clusters or clusters in different developmental contexts. All things considered, it offers an alternative conclusion and inspires us to communicate exceptional outcomes from contrary degrees.

Wang, Yang [22] analyze the influence of national high-tech zones on regional economic growth in China and use a panel data set from 281 prefecture-level cities spanning 1994 to 2019, adopting the differences-in-differences (DID) approach to estimate the impact. According to the study’s findings, national high-tech zones support regional economic growth, and multiple robustness tests support this conclusion. This article is identical to Hu, Huang [16] and Liu, Zhang [18], both of which apply the DID method. While the DID method is advantageous for accounting for unobserved heterogeneity and yielding a more precise estimate of the causal impact of high-tech zones, we think it may overlook differences in the dates of special economic zone foundation, which could lead to inaccurate findings.

Most similar to our findings, Zheng and Li [23] contend that the HTIDZ policy considerably increases cities’ capacity for innovation. The essay also argues in favor of the HTIDZ policy to improve collaboration across industry, academia, and government by attracting relevant firms from elsewhere. The fact that the ETDZ is part of the NLDZ broadens the scope of our study. If “pilot governance" is defined as HTIDZ, the outcome is incomplete. The NLDZ referred to in our paper as the Pilot Governance should comprise HTIDZ, ETDZ, TR, BA, and so on. The “China Development Zone Review and Announcement Catalogue (2018)" should serve as the foundation for the specific list.

Above all, the difference in our article is that we divide the NLDZ into categories based on their goals, such as ETDZ, HTIDZ, BA, TR, and so on. Then, we prefer to use panel data rather than the difference-in-differences method to examine cities’ innovation.

Moreover, Ziedina and Pelse [24] contend that a plethora of studies indicate that Latvia’s economic circumstances have improved and that to promote innovation and better growth, the government should devise a new plan for the construction of special economic zones. The authors discuss how to create a special economic zone and provide examples from developing nations using the methodology they have outlined. The paper validates the findings of our investigation and provides practical conclusions for future reference. These articles provide us with inspiration on the special economic zone from a variety of angles, and we make varying degrees of reference to them.

According to previous studies, the NLDZ will attract enterprises that collaborate and create scale economic effects. This effect reduces production costs and increases the opportunities for collaborative innovation. On the other hand, NLDZ development also attracts talent, and talent agglomeration enhances firms’ innovation efficiency. So, this article puts forward the Hypothesis 1:

*Hypothesis 1: The development of NLDZ will positively foster the innovation abilities of cities.*

## Methods

### Data

The dependent variable, *Number of Patents Granted (NPG)*, is from the “Chinese Urban Statistical Yearbook”. The explanatory variable, *Budget for General Public Revenue (BGPR)*, is sourced from public data on the NLDZ government website. The explanatory variable *Intellectual Property Filing (IPF)* is from the “Peking University Magic Weapon" - China Law Retrieval System. All other explanatory data come from the Chinese Urban Statistical Yearbook. The data covers the years 2014-2022. Considering this period, first, it covers the years since 2014, when the 541 NLDZ was built, and second, data for the latest year, 2023, are hard to obtain due to delays in the release of statistical yearbooks. There is a total of 541 NLDZ in the data, including 219 ETDZ, 164 HTIDZ, 130 BA, and 28 Others. The list of NLDZ is from the “China Development Zone Review and Announcement Catalogue (2018)”. However, there have been changes in the numbers in recent years, and some of the cities’ data is missing. The missing cities’ data include Honghe, Dali, Erlian Hot, Yanbian, Chuxiong, Dehong, Xishuangbanna, Haixi, Shihezi, Bayingguo Leng, Yili, Alal, Wujia, Canal, Changji, Aksu, Boltala, Kashta, Ta City, and Altai, for a total of 20 cities. If a value is missing, we perform linear interpolation; if the result is negative, we set it to 0. Then, all the data are incremented by 1 and then logarithmically transformed. To avoid the unbalanced panel data, we changed all the 0 variables to 0.01.

### Variables

#### Dependent variable.

We use NPGs to express each city’s innovation capacity; the more NPGs, the stronger the capacity.

#### Explanatory variables.

**Fig 1:**
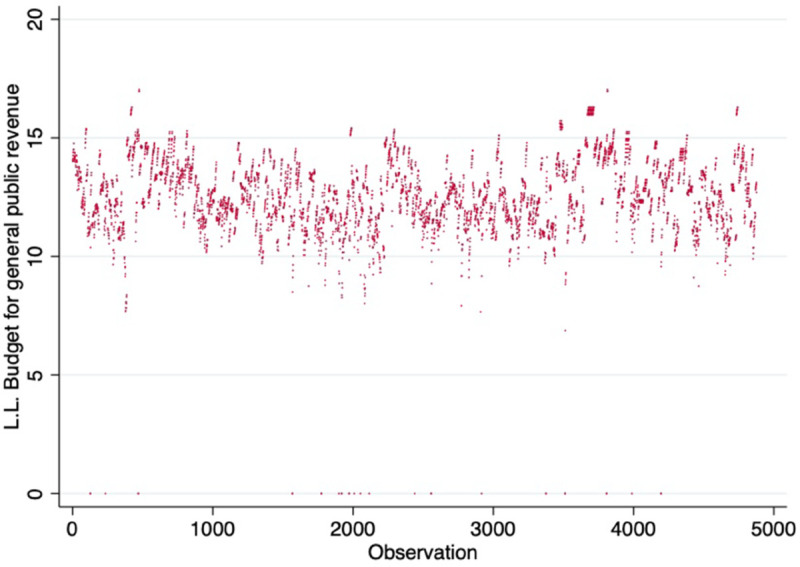
The distribution of BGPR.

Since BGPR indicates the amount of tax income each NLDZ will generate, we use it to measure the economic size of each NLDZ. An increase in tax revenue suggests an expansion in the NLDZ’s economic scale. Additionally, the study shows that cities’ innovation capacity increases with the size of their NLDZ economies. It is important to note that some BGPR data for NLDZ are missing, so we use the BGPR for the county where NLDZ is located as a substitute. Meanwhile, to avoid endogeneity and account for the lag in innovation output, we apply a 2-year lag for BGPR. From the Fig 1, we can see that most of the BGPRs are distributed between 10 and 15, except for a few missing values.

#### Control variables.

The impact of talent agglomeration on a city’s ability to innovate is expressed by the *Number of Ordinary Higher Education Students in School (NOHESS)*. The effect of R&D investment on a city’s ability to innovate is illustrated by *Expenditure on Science and Technology (EST)*, which is derived from a range of sources, including government, academic institutions, businesses, and others. Since innovation activities are closely related to intellectual property protection, this article uses IPF to capture the level of intellectual property protection across cities. To characterize the financial influence on cities’ capacity for innovation, we use *the Balance of Various RMB Deposits in Financial Institutions (BVDFI)*. The easier it is for the business to get bank loans, the higher the BVDFI. Lastly, we use GDP to show how cities’ innovation capacity is affected by economic growth. All these variables are from different cities where the NLDZ is located.

#### Describe variables.

The raw data for the variables are shown in Table 1. The data have been linked to this article, and Table 2 reports the normal distribution tests for the logarithmic variables.

**Table 1:** Describe variables.

Variable	Unit	Logarithm	Observations	Mean	SD	Minimum	Maximum
NPG	One	Yes	4,869	22,822	38,456	0	279,177
BGPR	10,000 Yuan (1,406$)	Yes	4,869	746,377	1,592,146	0	25,100,000
NOHESS	One	Yes	4,869	269,003	391,773	0	4,124,862
EST	10,000 Yuan (1,406$)	Yes	4,869	498,315	898,659	0	5,549,817
IPF	One	Yes	4,869	1,754	4,012	0	46,167
BVDFI	10,000 Yuan (1,406$)	Yes	4,869	167,000,000	281,000,000	1,470,727	2,120,000,000
GDP	10,000 Yuan (1,406$)	Yes	4,869	74,100,000	83,000,000	319,184	447,000,000

Notes: The data sources mentioned above refer to “3.1 Data”.

**Table 2:** Shapiro–Francia W’ test for normal distribution.

Variable	Obs	W’	V’	z	Prob>z
NPG	4,869	0.836	466.145	15.539	0.001
BGPR	4,869	0.813	531.973	15.873	0.001
NOHESS	4,869	0.889	315.323	14.550	0.001
EST	4,869	0.988	31.782	8.747	0.001
IPF	4,869	0.990	27.256	8.359	0.001
BVDFI	4,869	0.984	42.828	9.502	0.001
GDP	4,869	0.990	27.776	8.407	0.001

Notes: The Obs means the number of observations, W’ and V’ are various calculated values to justify whether it is belongs to normal distribution.

Table 1 describes the relevant variables. This article involves 9 years spanning and 541 NLDZ, so the observations are 4869. Given the substantial disparity in the data’s absolute values, the assumption of normality will not be met in empirical research. Therefore, it is necessary to perform a logarithmic transformation of the data to ensure the distribution approximates a normal distribution and reduce estimation errors. On the other hand, the logarithmic measure can reduce the influence of extreme observation values, which may otherwise bias the coefficient.

In the meantime, 11 of the total 552 NLDZ are missing due to prefecture-level data loss, and these cities are in less developed areas. The missing prefecture-level data is described above. As mentioned above, if the variables contain missing data, this article will use linear interpolation. The missing proportion of NPG is about 1/10, and the BGPR is about 1/100. The NOHESS, EST, and IPF are 3/1000, 4/10000, and 1/100, respectively. These missing values will be handled in several steps. First, we use linear interpolation to fill in these missing values. Second, if the interpolation result is negative, we change it to 0. Finally, after taking the logarithm of all the variables, due to log (0) having no meaning, we substitute it with 0.01 (Due to the presence of missing values, using linear interpolation is a commonly adopted practice. The linear interpolation method assumes that the data changes smoothly over a short period of time, and the method is relatively simple and easy to implement).

Table 2 presents the test for normality of logarithmic variables. The p-value is less than 0.01, indicating that the null hypothesis of normality is rejected; therefore, we conclude that the variables aren’t normally distributed. Even though the variables are not normally distributed, this will not affect the OLS hypothesis. However, the distribution of the residuals will impact the assumptions of the least squares method. The correlation analysis between variables is shown in Table 3.

**Table 3:** The correlation analysis between variables.

	NPG	BGPR	NOHESS	EST	IPF	BVDFI	GDP
NPG	1						
BGPR	0.410*** (0.000)	1					
NOHESS	0.377*** (0.000)	0.419*** (0.000)	1				
EST	0.521*** (0.000)	0.639*** (0.000)	0.700*** (0.000)	1			
IPF	0.532*** (0.000)	0.572*** (0.000)	0.680*** (0.000)	0.818*** (0.000)	1		
BVDFI	0.523*** (0.000)	0.630*** (0.000)	0.788*** (0.000)	0.894*** (0.000)	0.855*** (0.000)	1	
GDP	0.515*** (0.000)	0.644*** (0.000)	0.743*** (0.000)	0.909*** (0.000)	0.855*** (0.000)	0.954*** (0.000)	1

Notes: p < 0.1 +; p < 0.05 *; p < 0.01 **; p < 0.001 ***.

It’s evident that all variables in Table 3 are significantly correlated, so it’s necessary to utilize an econometric method to identify their causal relationship.

### Model

We then decide whether to use a fixed-effects or a random-effects model, since we are working with panel data. The results of Hausman’s test are more definitive, indicating that a fixed-effect model should be selected (Chi2(7) = 1628.82, p-value = 0.001). The joint significance of the annual dummy variables is tested after deciding whether to employ the two-way FE model, and the findings indicate that the two-way FE model should be chosen. There is a 0.001 p-value. The following is the model:

$$NPG_{it}=BGPR_{it}+NOHESS_{it}+EST_{it}+IPF_{it}+BVDFI_{it}+GDP_{it}+\mu_{i}+\gamma_{t}+\varepsilon_{it}$$ (1)

NPG is the dependent variable. BGPR is an independent variable. NOHESS, EST, IPF, BVDFI, and GDP are control variables. Where *t* stands for years and *i* for the city. $\mu_{i}$ describes the city’s fixed effect. $\gamma_{t}$ expresses *t* years fixed effect. $\varepsilon_{it}$ is a random disturbed term.

## Results

### Main results

The result is shown in [table4]Table 4. Baseline regression results (Column (1)) demonstrate that BGPR significantly increases cities’ innovation capacity. It indicates that cities’ innovation capacity will increase by 0.099% for every 1% increase in BGPR. We test the hypothesis H0 = 0 using an F test to decide whether to use fixed-effect or pooled regression. Next, we reject H0 because the p-value is less than 0.001. Fixed-effect regression is the preferred option. Column (2) is the fixed effect regression result. On the other hand, the mean Variance Inflation Factor (VIF) is 7.32, which is less than 10, indicating that multicollinearity is not severe enough to affect coefficient significance and that the results are reliable.

[p!]

**Table 4:** Main results.

*DV: NPG*	*Model*										
	(1)	(2)	(3)	(4)	(5)	(6)	(7)	(8)	(9)	(10)	(11)
BGPR	$0.099^{*}\;(0.046)$	$0.208^{***}\;(0.055)$	$0.253^{***}\;(0.066)$	$0.341^{***}\;(0.055)$	$0.157^{**}\;(0.050)$	$0.220^{***}\;(0.041)$	$0.259^{***}\;(0.079)$	$0.089^{*}\;(0.043)$	$0.135^{**}\;(0.065)$	$0.105^{***}\;(0.021)$	$0.105^{***}\;(0.010)$
NOHESS	0.037(0.040)	0.037(0.276)	$-0.399^{***}\;(0.087)$	$-0.844^{*}\;(0.390)$	0.043(0.043)	−0.026(0.264)	$-1.012^{*}\;(0.043)$	0.001(0.046)	0.001(0.127)	$-0.083^{+}\;(0.049)$	$-0.083^{**}\;(0.036)$
EST	$0.450^{***}\;(0.064)$	$0.313^{**}\;(0.108)$	$0.301^{***}\;(0.084)$	$0.432^{***}\;(0.135)$	$0.380^{***}\;(0.069)$	$0.272^{**}\;(0.111)$	0.139(0.102)	$0.265^{***}\;(0.069)$	$0.135^{**}\;(0.060)$	$0.066^{***}\;(0.019)$	$0.066^{***}\;(0.017)$
IPF	$0.181^{***}\;(0.041)$	−0.013(0.039)	$0.444^{***}\;(0.047)$	$0.474^{***}\;(0.041)$	$0.102^{**}\;(0.037)$	−0.005(0.038)	$0.200^{***}\;(0.025)$	$0.169^{**}\;(0.061)$	$0.318^{***}\;(0.024)$	$0.080^{***}\;(0.006)$	$0.080^{***}\;(0.006)$
BVDFI	0.063(0.160)	$1.098^{+}\;(0.584)$	$1.425^{***}\;(0.224)$	$7.063^{***}\;(0.638)$	$0.471^{**}\;(0.160)$	0.715(0.499)	$6.694^{***}\;(1.615)$	$0.681^{***}\;(0.109)$	$4.022^{***}\;(0.155)$	$0.946^{***}\;(0.107)$	$0.946^{***}\;(0.042)$
GDP	0.151(0.209)	$-1.612^{***}\;(0.376)$	$-0.839^{**}\;(0.269)$	$-1.232^{*}\;(0.580)$	−0.177(0.199)	$-0.900^{***}\;(0.243)$	−0.656(0.976)	0.055(0.073)	$-0.486^{**}\;(0.185)$	$-0.294^{**}\;(0.096)$	$-0.294^{**}\;(0.052)$
Logarithm	Yes	Yes	Yes	Yes	Yes	Yes	Yes	Yes	Yes	Yes	Yes
FE-city	No	Yes	No	Yes	No	Yes	Yes	No	Yes	Yes	Yes
FE-year	No	Yes	No	No	Yes	Yes	No	No	No	No	No
FE-region	Yes	Yes	No	No	No	No	No	No	No	No	No
N	4,869	4,869	4,869	4,869	4,869	4,869	4,869	4,869	4,463	4,869	4,869
R-sq	0.6318	0.6274	0.3117	0.2932	0.6281	0.5967	0.1828	0.5270	0.5392	None	None

*Notes:*  ^+^ *p*<0.1; *p < 0.05; **p < 0.01; ***p < 0.001. The coefficient is shown on the first line, and the standard error on the second.

At the 0.001 level, Column (2) indicates that a 1% increase in BGPR will result in a 0.208% increase in a city’s capacity for innovation. It argues that the creation of NLDZ will significantly increase cities’ capacity for innovation. Every 1% increase in NOHESS will boost cities’ innovation capabilities by 0.037%; however, the association is not significant. The most likely explanation is that as more elite cities remove the residence requirement, an increasing number of students are choosing to live elsewhere rather than in their hometowns [25]. The innovation capacity of cities will rise by 0.313% at the 0.01 level with a 1% EST increase. The coefficient for EST indicates that greater investment in R&D is associated with a city’s greater innovation capacity. The coefficient of IPF is negative and not significant. A 1% increase in IPF will decrease cities’ innovation capabilities by 0.01%. The association between the two variables is not statistically significant, and the negative coefficient for IPF indicates that the innovation protection system in our nation is incomplete [26]. Because of its minimal coefficient, the IPF has little economic significance. According to the BVDFI coefficient, cities’ capacity for innovation will grow by 1.098% at the 0.01 level for every 1% increase in BVDFI. This finding indicates that more lenient bank lending policies will motivate more businesses to invest in research and development. Finally, GDP severely limits cities’ capacity to drive economic outcomes. However, we believe there is an endogenous flaw in the relationship between GDP and a city’s capacity for innovation, such as mutual causality, which makes the result inaccurate.

### Robustness test

We completed some of the work indicated in Columns (3) through (8) for the robustness test. We apply a random effect to the panel data processing in Column (3). Instead of using a two-way fixed effect, we manipulate the FE-year and FE-city in Columns (4) and (5), respectively. We replace the explanatory variable BGPR in Column (6) with *the Tax Revenue of NLDZ (TRNLDZ)*. Similarly, we take TRNLDZ’s two-year lag and logarithm it using base *e*. We substitute *GDP per capita (GDPPC)* for one of the control variables, GDP, in the interim. Instead of using FE to estimate the result, we use the First-Order Differencing Estimator in Column (7). Then, we change the dependent variable. *The Number of Invent Patents Granted (NIPG)* is replaced with NPG, and TRNLDZ and GDPPC are used in place of BGPR and GDP, respectively. In Column (8), we do not utilize FE but rather the Between Estimator; the result is shown in Column (8).

In Column (9), this article uses Cook’s distance to evaluate robustness. First, it’s necessary to calculate the Cook’s distance for each observation; then, this article deleted 406 abnormal or high-leverage observations that exceeded the Cook’s distance limit. Finally, this article still uses panel data with fixed effects, and the results are shown in Column (9). The BGPR still has a significant impact on the NPG. The result is robust.

In Column (10), this article uses Poisson regression with an individual fixed effect to estimate the count-dependent variable. To mitigate heteroscedasticity, this model uses bootstrap standard errors. The results show that the significance of BGPR remains robust. The coefficient of BGPR is slowly less than that of Model 3, even though these two coefficients cannot be compared with each other, because the meaning of the Poisson regression coefficient is different from the OLS results.

This article takes Negative Binomial regression with individual fixed effect in Column (11). As shown above, the coefficient of Model 11 is close to that of Model 10, but the standard error is different from that of Model 10. The coefficient for BGPR remains significant at the 0.001 level, indicating robustness.

Following the aforementioned robustness test, all data indicate that BGPR is strongly associated with cities’ innovation capacity, validating our hypothesis that NLDZ will foster it.

### Endogenous problem

#### Reverse causality.

As shown above, the development of NLDZ has a positive effect on cities’ innovation ability. But there is another possibility: as cities’ innovation capacity increases, this zone will attract more powerful enterprises to locate in, and these enterprises contribute to the general public revenue. This phenomenon is called reverse causality. To address this problem, as previously noted, we use a 2-year lag in BGPR to mitigate endogeneity. This measure means that the Lag of BGPR may cause a change in NPG, but the NPG is hard to affect the two-year lag of BGPR due to time lags.

#### Omitted variables bias.

Omitted variables in the model will create an endogeneity problem. To prevent endogenous issues caused by missing variables, we use fixed effects of year and city.

#### Instrumental variables.

Even though this article uses fixed effects to avoid omitted-variable bias, unobserved factors still affect the model, leading to endogeneity. Therefore, we adopt a one-year lag of BGPR, L2. BGPR and L3. BGPR, as instrumental variables of BGPR (Lag one-year of BGPR, L2. BGPR and L3. BGPR is actually the three-year lag of current year of BGPR, four-year lag of current year of BGPR and five-year lag of current year of BGPR, respectively). The L1. BGPR, L2. BGPR and L3. BGPR all are relevant to BGPR on the one hand, but it does not affect the NPG because these variables occurred before the NPG. The result is shown in Table 5.

**Table 5:** Instrumental variables test.

Variables	BGPR	NPG
	(1)	(2)
L1. BGPR	$0.074^{*}\;(0.033)$	
L2. BGPR	−0.048(0.033)	
L3. BGPR	$0.063^{*}\;(0.030)$	
BGPR		$0.147^{**}\;(0.056)$
NOHESS	−0.001(0.041)	$0.445^{***}\;(0.048)$
EST	$0.056^{**}\;(0.018)$	$0.083^{***}\;(0.022)$
IPF	0.007(0.005)	$0.018^{**}\;(0.007)$
BVDFI	$-0.377^{***}\;(0.108)$	$1.413^{***}\;(0.060)$
GDP	$0.767^{***}\;(0.112)$	$0.167^{**}\;(0.072)$
Logarithm	Yes	Yes
FE-city	Yes	Yes
FE-year	No	No
N	1,623	3,246
R-sq	0.6686	0.7894
Test of overidentifying restrictions: Sargan-Hansen statistic: 3.005.		
Chi-sq (2) P-value = 0.2226.		

*Notes:*  ^+^ *p*<0.1; *p < 0.05; **p < 0.01; ***p < 0.001.

As shown in Table 5, the instrumental variables pass the overidentifying test; therefore, we cannot reject the null hypothesis that all instrumental variables are valid. The Model (1) is the first step result of the instrumental variables test, and the Model (2) is the second step result. It can be seen that BGPR has significantly affected the NPG. The result is robustness.

### Mechanism test

**Fig 2:**
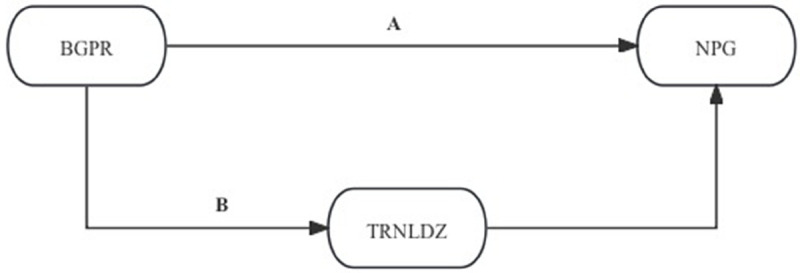
Mechanism TRNLDZ.

**Fig 3:**
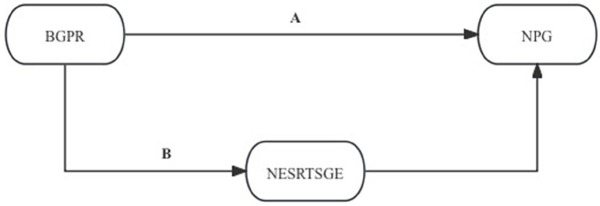
Mechanism NESRTSGE.

More specifically, we postulate that there are two paths by which BGPR influences NPG to illustrate the conduction pathway better. One path is through TRNLDZ to conduct NPG; the other is through *the Number of Employees in Science Research, Technical Services, and Geological Exploration (NESRTSGE)*. The national Bureau of Statistics combines SR, TS and GE together to form the NESRTSGE statistics in Chinese Urban Statistical Yearbook. The two paths are shown in Figs 2 and 3.

Fig 2 illustrates how BGPR influences TRNLDZ before conducting NPG in addition to its direct effect on NPG. Fig 3 shows that BGPR not only affects NPG on its own but also NESRTSGE, which in turn affects NPG. The mechanism test results are shown in Table 6.

**Table 6:** Mechanism test.

Variables	TRNLDZ	NPG	NESRTSGE	NPG
	(1)	(2)	(3)	(4)
BGPR	$0.916^{***}\;(0.007)$	$0.624^{***}\;(0.069)$	$-0.014^{*}\;(0.007)$	$0.171^{***}\;(0.029)$
TRNLDZ		$0.517^{***}\;(0.079)$		
NESRTSGE				$-0.397^{+}\;(0.205)$
BGPR*TRNLDZ		$-0.053^{***}\;(0.004)$		
BGPR*NESRTSGE				$-0.047^{**}\;(0.015)$
NOHESS	$0.034^{***}\;(0.010)$	$-0.291^{***}\;(0.043)$	$0.233^{***}\;(0.010)$	0.026(0.044)
EST	$0.106^{***}\;(0.013)$	$0.461^{***}\;(0.057)$	−0.013(0.014)	$0.353^{***}\;(0.056)$
IPF	$-0.014^{+}\;(0.008)$	$0.415^{***}\;(0.036)$	$-0.039^{***}\;(0.009)$	$0.393^{***}\;(0.035)$
BVDFI	$-0.056^{*}\;(0.027)$	$0.844^{***}\;(0.115)$	$1.008^{***}\;(0.028)$	$1.675^{***}\;(0.128)$
GDP	0.019(0.032)	$-0.289^{*}\;(0.134)$	$-0.135^{***}\;(0.033)$	$-0.549^{***}\;(0.132)$
Logarithmic	Yes	Yes	Yes	Yes
N	4,869	4,869	4,869	4,869
R-squared	0.8696	0.3356	0.8105	0.3577

^+^ *p*<0.1; *p < 0.05; **p < 0.01; ***p < 0.001. The symbol   denotes interaction terms.

Columns (1) and (3) are the first step of the mechanism test of Channel B and Channel A, respectively. It’s observed that BGPR contributes to the TRNLDZ in Column (1) and NESRTSGE in Column (3). Therefore, BGPR significantly affects the mechanism variables TRNLDZ and NESRTSGE. Columns (2) and (4) show that the mechanism variables are still valid when the BGPR is controlled due to TRNLDZ in Column (2) and NESRTSGE in Column (4) being significant to affect NPG. Besides, BGPR*TRNLDZ and BGPR*NESRTSGE are both significant and negative, indicating that these interaction effects negatively affect cities’ innovation capacity. The likely reason is that the NLDZ’s economic scale and R&D activities are prominent within the city’s overall economy. This single industrial chain may squeeze out innovation in other industrial chains. So, it reflects the negative effect on NPG. As a result, this article thinks the mechanisms are effective.

### Heterogeneity test

East, Middle, West, and Northeast are the four regions into which we separated the panel data in accordance with China’s regional development strategy [26,27]. Because every area has experienced unique problems and has adopted unique solutions [27]. In addition, we divided the panel data into two categories: prefecture-level cities and provincial cities, with municipalities included in the latter. The abolition of the household registration system has attracted many talented people to provincial cities, leading to a disparity in NLDZ development between prefecture-level and provincial cities. Ultimately, we separated the panel data into four categories: HTIDZ, BA, ETDZ, and others. The NLDZ comprises these four components. The outcomes are displayed in Tables 7 and 8.

**Table 7:** Heterogeneity tests I.

DV: Number of Patents Granted	Heterogeneity Test					
	Regional Heterogeneity (1)				Administrative Level (2)	
	East	Middle Part	West	Northeastern	Provincial-Level	Prefecture-Level
BGPR	$0.297^{***}\;(0.052)$	0.044(0.083)	$0.195^{**}\;(0.067)$	0.047(0.056)	$0.069^{+}\;(0.039)$	$0.095^{***}\;(0.026)$
NOHESS	$-0.668^{+}\;(0.034)$	$-1.577^{**}\;(0.352)$	$2.445^{***}\;(0.304)$	$0.875^{+}\;(0.457)$	$0.780^{***}\;(0.109)$	0.056(0.036)
EST	$1.092^{***}\;(0.186)$	$-0.789^{***}\;(0.188)$	$0.459^{**}\;(0.151)$	0.071(0.161)	$0.206^{+}\;(0.119)$	$0.299^{***}\;(0.054)$
IPF	$0.180^{*}\;(0.070)$	$-0.107^{*}\;(0.057)$	−0.101(0.062)	$0.153^{*}\;(0.074)$	$-0.301^{***}\;(0.058)$	$0.209^{***}\;(0.033)$
BVDFI	$3.799^{***}\;(0.757)$	0.844(1.126)	−0.874(1.153)	$-1.635^{+}\;(0.900)$	$-0.603^{**}\;(1.204)$	$0.646^{***}\;(0.123)$
GDP	$-4.632^{***}\;(0.478)$	$2.318^{***}\;(0.649)$	$-4.238^{***}\;(0.599)$	0.785(0.610)	$1.922^{***}\;(0.220)$	$-0.562^{***}\;(0.120)$
Logarithmic	Yes	Yes	Yes	Yes	Yes	Yes
FE-city	Yes	Yes	Yes	Yes	No	No
FE-region	Yes	Yes	Yes	Yes	Yes	Yes
FE-year	Yes	Yes	Yes	Yes	Yes	Yes
Balanced panel	Yes	Yes	Yes	Yes	Yes	Yes
N	2,371	1,070	930	463	1,512	3,357
R-squared	0.6989	0.7628	0.7995	0.8236	0.6989	0.7275
Coefficient difference test: Regional heterogeneity - F (3, 4821) = 5.07; Prob > F = 0.0017						
Administrative level - F (1, 4861) = 60.54; Prob > F = 0.000.						

^+^ *p*<0.1; *p < 0.05; **p < 0.01; ***p < 0.001. The first line represents the coefficient, and the second line represents the standard error.

**Table 8:** Heterogeneity tests II.

Variables	Heterogeneity Test by Zone Type (3)			
	Development Zone Type			
	ETDZ	HTIDZ	BA	Others
BGPR	$0.076^{*}\;(0.038)$	$0.075^{+}\;(0.038)$	$0.143^{***}\;(0.043)$	−0.045(0.206)
NOHESS	$0.101^{+}\;(0.053)$	0.034(0.075)	$0.180^{**}\;(0.059)$	0.242(0.177)
EST	$0.352^{***}\;(0.080)$	$0.469^{***}\;(0.083)$	$0.498^{***}\;(0.108)$	0.024(0.238)
IPF	$0.128^{**}\;(0.046)$	$0.138^{**}\;(0.048)$	$0.153^{*}\;(0.068)$	$0.281^{*}\;(0.129)$
BVDFI	0.074(0.146)	0.064(0.178)	$0.335^{+}\;(0.188)$	−0.480(0.468)
GDP	0.241(0.157)	0.154(0.171)	$-0.616^{**}\;(0.233)$	$1.368^{**}\;(0.428)$
Logarithmic	Yes	Yes	Yes	Yes
FE-year	Yes	Yes	Yes	Yes
FE-region	Yes	Yes	Yes	Yes
Balanced panel	Yes	Yes	Yes	Yes
N	1,971	1,476	1,170	252
R-squared	0.6156	0.6280	0.6445	0.7435
Coefficient difference test: F (3, 4856) = 0.73; Prob > F = 0.5329.				

^+^ *p*<0.1; *p < 0.05; **p < 0.01; ***p < 0.001. The coefficient is shown on the first line, and the standard error on the second.

Table 7 Column (1) demonstrates that there are differences in the impact of NLDZ on cities’ capacity for innovation. The NLDZs in the East and West have greater effects on urban innovation than the other NLDZs, whereas the NLDZs in the Middle Part and Northeast have the least impact, and the coefficients for the Middle Part and Northeast are not significant. The most likely explanation is that Middle Part and Northeastern cities mostly rely on natural resources for their economic growth, which has minimal bearing on urban innovation [28].

Table 7 Column (2) shows that NLDZ has an impact on urban innovation, no matter whether province- or prefecture-level cities. Meanwhile, the coefficient of Province-Level is less than that of Prefecture-Level. The reason is that more talented people agglomerate in province-level cities because there are more job opportunities there than in other places. Because of a larger economic scale, the NLDZ’s marginal effect at the province-level is less than at the prefecture-level cities. To confirm the difference in the coefficient, this article uses dummy variables to test it. The result is shown in the last row of Table 7. The result shows a significant difference in the BGPR coefficient and supports the heterogeneity test.

Table 8 demonstrates the diverse effects of the various NLDZ types. The BA has the most considerable influence on urban innovation, followed by ETDZ and HTIDZ. The Others have the least impact. Meanwhile, the Others’ coefficient is not significant, most likely because of the resource- and labor-intensive nature of the TR and BECZ businesses (Others include TR and BECZ). Thus, the impact on urban innovation is not readily apparent. The coefficient difference test is not significant, indicating that we can’t reject the null hypothesis of no significant difference in the BGPR coefficient. One probable reason is the convergence of policy effects. Whether the zone type differs or not, the NLDZ fosters cities’ innovation capacity through knowledge spillovers, agglomeration effects, and infrastructure sharing. In this process, the policy interaction is similar across zone types.

### Impact of NLDZ quantity

**Fig 4:**
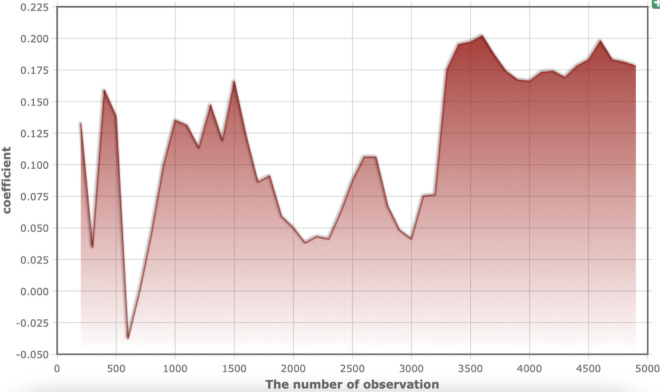
Impact of NLDZ quantity.

We would like to know how much NLDZ is appropriate or whether more NLDZ is better, given the rise in NLDZ in China. Thus, we regress the data several times in a random order using the available data. The outcome is displayed in Fig 4.

The regression coefficient is shown on the vertical axis, and the number of observations is shown on the horizontal axis. Fig 4 shows that the coefficient varies from low to high as the number of NLDZ increases. It implies that while a certain amount of NLDZ will increase influence and foster urban innovation, an excessive amount will have a diminishing marginal effect.

### Sensitivity analysis

This article adds random errors to NPG and tests whether BGPR still significantly affects NPG. The generation of random errors is as follows,

$$Y_{new}=Y_{original}+k*\sigma_{y}*\varepsilon$$ (2)

Where $Y_{\text{new}}$ represents the NPG with random errors being added deliberately. $Y_{\text{original}}$ is the original NPG. *k* describes the coefficient, and it takes 0.05, 0.1, and 0.2 in this article. $\sigma_{y}$ represents the standard error of the dependent variable. *ε* is a random coefficient extracted from a standard normal distribution. This article regresses the model, runs 1,000 simulation tests, and then presents the sensitivity test results in Table 9.

**Table 9:** Sensitivity test.

Degree of Random Error	Mean Coefficient	Std. Error	Sig. at 0.05	Sig. at 0.10
5%	0.1777	0.0019	1.0000	1.0000
10%	0.1777	0.0037	1.0000	1.0000
20%	0.1776	0.0072	1.0000	1.0000

*Notes:* The null hypothesis is that there are no substantive changes after adding the random error.

It’s observed that the coefficient’s mean value remains nearly unchanged across different levels of random error, and the significance level is greater than 0.01. So, this article can’t reject the null hypothesis. Therefore, this article argues that BGPR continues to affect NPG significantly and that the results are robust.

Besides, this article substitutes BGPR to test the robustness of the results. In Column (6) and Column (8) of [table4]Table 4, we substitute TRNLDZ for BGPR, and the coefficient is also significant. Above all, the result is robustness.

### Stepwise regression

In this section, the article adds the control variables to the main result step by step, then examines the BGPR coefficient to NPG. The result is shown in Table 10.

**Table 10:** Stepwise regression.

DV: NPG	Model					
	(1)	(2)	(3)	(4)	(5)	(6)
BGPR	$0.855^{***}\;(0.069)$	$0.700^{***}\;(0.062)$	$0.556^{***}\;(0.050)$	$0.507^{***}\;(0.055)$	$0.328^{**}\;(0.056)$	$0.341^{***}\;(0.055)$
NOHESS		$4.671^{***}\;(0.388)$	$3.395^{***}\;(0.384)$	$3.049^{*}\;(0.375)$	$-0.928^{*}\;(0.404)$	$-0.843^{*}\;(0.390)$
EST			$1.178^{***}\;(0.203)$	$1.453^{***}\;(0.183)$	$0.248^{+}\;(0.147)$	$0.432^{***}\;(0.135)$
IPF				$0.779^{***}\;(0.053)$	$0.479^{***}\;(0.041)$	$0.473^{***}\;(0.041)$
BVDFI					$6.425^{**}\;(0.395)$	$7.062^{***}\;(0.638)$
GDP						$-1.232^{*}\;(0.580)$
Logarithm	Yes	Yes	Yes	Yes	Yes	Yes
FE-city	Yes	Yes	Yes	Yes	Yes	Yes
FE-year	No	No	No	No	No	No
N	4,869	4,869	4,869	4,869	4,869	4,869
R-squared	0.0794	0.1920	0.2656	0.3265	0.4770	0.4793

^+^ *p*<0.1; *p < 0.05; **p < 0.01; ***p < 0.001. The coefficient is shown on the first line, and the standard error on the second.

**Fig 5:**
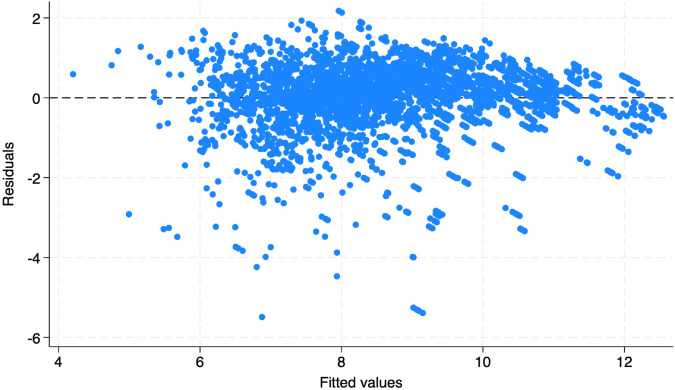
Residual plots.

As shown in Table 10, when adding the control variables, the BGPR decreases stepwise until it reaches 0.3 in Column (6). The significance of BGPR remains at the 0.001 level. Moreover, all control variables remain significant for cities’ innovation capacity, and the results demonstrate the robustness of the regression model. Finally, the residual plot is shown in Fig 5.

As shown in Fig 5, the residuals are centered around 0, with a few being negative. Therefore, this article argues that the residuals are random and that the OLS assumptions are valid.

## Discussions

### Significance of special economic zones

The findings above indicate that the creation of NLDZ will greatly enhance cities’ capacity for innovation. The government spends a lot more money developing a place that attracts relevant industrial enterprises that cluster there [29]. Concurrently, the government established innovation hubs in the region to support businesses and develop skilled workers [30,31]. Each of these actions contributes to the agglomeration effect and strengthens cities’ capacity for creativity [32]. It is important to recognize that despite the NLDZ’s apparent benefits, there are still specific problems with its construction. First, the lack of digital tools for production process management in NLDZ firms will impede the shift to a digital economy [33]. Second, the government spends a significant amount of money building the NLDZ. To prevent wasteful spending, the government should establish a system to evaluate how the funds are being used [34,35]. Third, the development of small- and medium-sized high-tech businesses should be a government priority. These businesses typically have unique technologies and will generate more revenue in the future, even though they are unable to provide significant tax revenue in the near term [36].

### Employees in R&D

The mechanism test indicates that the number of workers in R&D will directly impact the capacity for urban innovation. It is essential to raise staff motivation in R&D. Many cities today implement various talent-attracting policies; nevertheless, we think the government should take a longer-term approach rather than pursuing a short-term strategy [37,38]. For instance, the government ought to provide high-quality, comfortable public residential amenities for housing, senior care, education, and transportation, among other things [39]. The government should encourage industry-university-research cooperation because many patents are made in conjunction with scientific institutions and businesses [40]. One way to do this would be to enhance higher education programs to better meet the demands of businesses [41]. Furthermore, local governments must improve intellectual property laws and enforcement [42,43].

### Heterogeneity discussion

#### Various geographical areas.

We might infer from Table 7 Column (1) that the East experiences the most tremendous NLDZ impact, with the West following suit. These patterns reflect differences in the stages of economic development between East and West. China’s most innovative region is the East, which produces the greatest innovations overall [44]. The businesses gathered here exhibit innovation and breakthrough qualities and have made the most considerable contributions to China’s economic expansion [45]. Rich in renewable energy resources, the West area serves as a major hub for the Belt and Road Initiative and is home to numerous large science instruments [46,47]. Thus, the industries associated with NLDZ improve the city’s capacity for innovation. Nonetheless, there is very little influence of the Northeast and Middle Parts’ NLDZ on urban innovation. The probable reason is that numerous creative businesses are departing these locations due to resource depletion and skill talent loss [48]. The remaining resource-intensive companies contribute significantly to economic growth, but their impact on cities’ capacity for innovation is little [49]. To address these problems, we should exploit resources in an orderly manner and improve resource utilization through science and technology [50,51]. Second, locals should seek alternative industries to replace resource-intensive ones, leveraging the area’s advantages.

#### Cities in provinces and prefectures.

Table 7 Column (2) indicates that the provincial city experiences a less impact from NLDZ compared to the prefecture-level city. The most likely explanation is that, as household registration is relaxed, a large number of creative businesses and talented individuals are moving to provincial cities, where they are clustering in knowledge-intensive areas like NLDZ [48]. Therefore, a provincial city’s capacity for innovation will grow faster than that of a prefecture city and have diminishing marginal utility. In light of these conditions, the government should support provincial cities’ ability to spur innovation. In contrast, prefecture-level cities should leverage their current resources to strengthen their relationships with provincial cities [51].

#### Various NLDZ kinds.

Table 8 shows that the effect of BA is the greatest, followed by that of ETDZ, while the impact of HTIDZ is the least. The BA was founded relatively late and has a small economic scale, which is likely the cause (Data from the official websites of the Ministry of Commerce and the Ministry of Science and Technology). The notion of diminishing marginal utility suggests that its potential contribution to innovation is highest. Because of diminishing marginal value, HTIDZ has a larger economic scale than ETDZ, so it has a smaller impact on urban innovation. It is found that a massive economic scale and quantity of NLDZs have a diminishing marginal effect on urban innovation, as shown in the analysis above.

#### A specific quantity of NLDZ.

From Fig 4, we hope to control the number of NLDZ reasonably. The China Development Zone Review and Announcement Catalogue (2018) states that there are 552 in total. The development of NLDZ should take measures for rewards and punishments and suggest specific fixes for current issues, but we do not advocate for the continuous expansion of NLDZ.

## Conclusion

The article discusses how the National Level Development Zone’s development has affected urban innovation using panel data from 2014 to 2022. Compared to the West, the East NLDZ has a greater effect. The NLDZ in the Northeast and the Middle Part has the least influence on urban innovation. The impact of a city at the province level is less than that of a city at the prefecture level, and the effect of BA is the greatest, followed by that of ETDZ, while the impact of HTIDZ is the least. Finally, we advise preserving a specific quantity of NLDZ to prevent the effect of declining marginal utility.

However, there are some obvious weaknesses, too, and we expected readers to continue our research. During the study, we use a linear interpolation method for some missing years, but these data can be obtained from internal government sources to improve accuracy. Meanwhile, not all 552 NLDZ are fully covered due to data limitations, and the Tax Revenue of NLDZ is more suitable for reflecting economic scale. Still, the data in this article are incomplete. Meanwhile, classifying and evaluating the innovation efficiency of national development zones and exploring spillover effects between national-level development zones can be the next research direction. Naturally, there are still many areas that need improvement, and we look forward to the outcomes of the upcoming studies.

## Acronym

Bond Area (BA)

Budget for General Public Revenue (BGPR)
Deposits in Financial Institutions (BVDFI)
differences-in-difference (DID)
Economic and Technological Development Zone (ETDZ)
Expenditure on Science and Technology (EST)
GDP per capita (GDPPC)
High-Tech Industrial Development Zone (HTIDZ)
Intellectual Property Filing (IPF)
National Border Economic Cooperation Zone (BECZ)
National Level Development Zone (NLDZ)
Number of Employees in Science Research, Technical Services and Geological Exploration (NESRTSGE)
Number of Invent Patents Granted (NIPG)
Number of Ordinary Higher Education Students in School (NOHESS)
Number of Patents Granted (NPG)
Tax Revenue of NLDZ (TRNLDZ)
Tourism Resort (TR)
Variance Inflation Factor (VIF)

## Supporting information

S1 file This file contains the STATA command.
(ZIP)

S2 file This file contains the data of the article.
(ZIP)
